# Cardioprotective Effects of *Solidago microglossa* DC. in Nicotine-Treated Hypertensive Rats

**DOI:** 10.1155/2023/6611569

**Published:** 2023-12-22

**Authors:** Katiana Simões Lopes, Aline Aparecida Macedo Marques, Karyne Garcia Tafarelo Moreno, Ariany Carvalho dos Santos, Roosevelt Isaías Carvalho Souza, Emerson Luiz Botelho Lourenço, Rodrigo Juliano Oliveira, Roberto da Silva Gomes, Francislaine Aparecida dos Reis Lívero, Arquimedes Gasparotto Junior

**Affiliations:** ^1^Laboratory of Cardiovascular Pharmacology (LaFaC), Faculty of Health Sciences, Federal University of Grande Dourados (UFGD), Dourados, MS, Brazil; ^2^Laboratory of Natural Products, Paranaense University (UNIPAR), Umuarama, PR, Brazil; ^3^Centro de Estudos em Células Tronco, Terapia Celular e Genética Toxicológica (CeTroGen), Faculdade de Medicina (FAMED), Universidade Federal de Mato Grosso do Sul (UFMS), Campo Grande, Mato Grosso do Sul, Brazil; ^4^Department of Pharmaceutical Sciences, North Dakota State University, Fargo, North Dakota 58102, USA; ^5^Laboratory of Cardiometabolic Pharmacology, Federal University of Paraná, Curitiba, Brazil; ^6^Laboratory of Preclinical Research of Natural Products, Paranaense University, Umuarama, Brazil

## Abstract

*Solidago microglossa* DC. (Asteraceae), “arnica brasileira,” is a Brazilian species popularly used to treat hypertension or renal ailments. This study investigated the cardioprotective effects of standardized *S. microglossa* extract (EESM) in nicotine-treated spontaneously hypertensive rats (SHRs). Moreover, the molecular mechanisms involved in the cardiovascular effects were also investigated. The acute toxicity was evaluated in female Wistar rats. Afterwards, six-month-old male spontaneously hypertensive rats received the EESM (14, 28, and 56 mg/kg), hydrochlorothiazide (25 mg/kg), and vehicle (filtered water; 0.1 mL/100 g) once daily for 28 days. All treatments were associated with 1.8 mg/kg of nicotine. At the end of the experimental period, the renal function, electrocardiographic profile, blood pressure, ventricular function, biochemical parameter, and mesenteric vascular bed reactivity were evaluated. Relative organ weights and cardiac morphometry were also investigated. Nicotine treatment in 6-month-old SHRs induced a significant reduction in renal function, with reduced urinary volume and lower renal elimination of sodium and creatinine. In addition, serum markers of the redox state and blood pressure levels remained significantly elevated, contributing to changes in vascular reactivity and left ventricular hypertrophy associated with reduced ventricular function. After 28 days of treatment, we found that the highest dose of EESM could mitigate all renal and cardiovascular changes developed by the nicotine-treated hypertensive rats. This study presented EESM as a possible cardioprotective drug that prevents cardiovascular dysfunctions in nicotine-treated hypertensive rats. Our data suggest EESM as a potential adjuvant therapy when cardioprotective effects are required.

## 1. Introduction

Hypertension is a multifactorial and highly prevalent disease considered to be the leading risk factor for developing several cardiovascular and renal diseases. The treatment includes lifestyle changes and pharmacological measures, including renin-angiotensin system inhibitors, beta-blockers, calcium channel blockers, and diuretics [1]. The drug therapy is lifelong and may cause several adverse effects, contributing to low adherence and inadequate disease control [2]. So, natural products emerge as an alternative to complementary and alternative therapy [3].

Due to Brazil's diversity of medicinal plants, public politics has encouraged projects and research to develop new herbal medicines. In 2009, a list of medicinal plants of interest to the Brazilian Unified Health System (SUS) was published, including *Solidago microglossa* DC. (Asteraceae) [4].

Popularly known as “arnica brasileira” or “arnica do mato,” *S. microglossa* is native to South America. The infusion from its leaves is popularly used to treat pain, inflammations, gastric disturbance, kidney diseases, hypertension, and constipation [5]. Pharmacological studies showed anti-inflammatory [6], antinociceptive [7], antischistosomal [8], and antimicrobial [9] activities. Chemical studies carried out with the crude extract identified the presence of quinic acid, quercetin, chlorogenic acid, hyperoside, rutin [6], baurenol, *α*-amirin, and spinasterol [8]. In addition, the essential oil from *S. microglossa* leaves presented *α*-pinene, *β*-pinene, myrcene, *α*-phellandrene, *δ*-3-carene, limonene, *β*-ocimene, acetophenone, *α*-pinene oxide,*α*-campholenal, dill ether, sesamol, isolongifolene, *α*-1,7-di-epi-cedrene, caryophyllene, *α*-humulene, *β*-cubebene, bicyclogermacrene, *α*-farnesene, *δ*-cadinene, spathulenol, 1,10-di-epi-cubenol, *α*-epi-cadinol, cubenol, *α*-eudesmol, and dihydroeudesmol [9].

Therefore, this work evaluated the cardioprotective effects of *S. microglossa* extract in nicotine-treated spontaneously hypertensive rats (SHRs). Moreover, the molecular mechanisms involved in the cardiovascular effects were also investigated.

## 2. Materials and Methods

### 2.1. Drugs, Salts, and Solutions

The following drugs and salts were used: ketamine hydrochloride, xylazine hydrochloride (Syntec, São Paulo, SP, Brazil), heparin (Hipolabor Pharmaceuticals, Belo Horizonte, MG, Brazil), phenylephrine (Phe), sodium nitroprusside (SNP), hydrochlorothiazide (HCTZ), angiotensin I, angiotensin II, endothelin 1, acetylcholine chloride (ACh), indomethacin, NaCl, KCl, CaCl_2_, MgSO_4_, NaHCO_3_, KH_2_PO_4_, dextrose, and ethylenediaminetetraacetic acid (EDTA) (Sigma-Aldrich, St. Louis, Missouri, USA). All other reagents were obtained with analytical grade.

### 2.2. *Solidago microglossa* Extract (EESM) Obtention

*S. microglossa* vegan capsules (Brazilian Arnica) were obtained from a Brazilian commercial preparation (Curitiba, Paraná, Brazil). Each capsule contains 650 mg of Brazilian Arnica dry extract. The extract was produced from the dried aerial parts of *S. microglossa* using 80–85% (v/v) ethanol. In the drying process, cornstarch was added at a ratio of 0.5–1.5 : 1. The dry extract was standardized at 1% quercetin-3-rhamnoside. Each capsule (composed of hypromellose and titanium dioxide) has a total weight of 1 g.

### 2.3. Pharmacological Investigations

#### 2.3.1. Animals

Male Wistar-Kyoto (WK) and male SHRs (180 days old), as well as female Wistar rats (90 days old), were obtained from the central vivarium of the Federal University of Grande Dourados (UFGD, Brazil). All animals were kept in a temperature and light-controlled room (22 ± 2°C; 12 h light/dark cycle) with ad libitum access to water and food. All procedures were previously approved by the Ethics Committee in Animal Experimentation from the UFGD (protocol no. 31/2019-1) [10, 11] and were following the Brazilian Legal Framework on Scientific Animals Use.

#### 2.3.2. Safety Evaluation

*(1) Acute Toxicity*. The acute toxicity assay was evaluated according to protocols from Palozi et al. [12]. Twelve female Wistar rats were equally randomized into two experimental groups. Initially, all animals were subjected to overnight fasting. Then, the animals were weighed, and a 2,000 mg/kg limit dose of EESM or vehicle (filtered water; 0.2 mL/100 g) was administered orally (by gavage). The rats were carefully observed for the first 8 hours and daily for 14 days. Mortality rate, water and food consumption, body weight gain, and any signs of toxicity or behavior changes were registered daily. On the fifteenth morning, all rats were killed by isoflurane anesthesia (inhalation) followed by exsanguination. Lungs, liver, kidneys, spleen, and heart were subjected to gross pathology.

#### 2.3.3. Cardioprotective Evaluations

*(1) Allometric Extrapolation of the EESM Daily Dose Used in Humans to Rats*. The EESM doses were determined by allometric extrapolation from the daily dose used for humans (1 g/day: 1 capsule). The average body weight considered for humans was 70 kg. Thus, the daily dose considered was 14, 28 mg/kg.

The metabolic rate of reference animal (MRref − human): MRref = *k* × *m* − 0.25 = 70 × 80 − 0.25 = 24.

The metabolic rate of target animal (MRtarget − rat): MRtarget = *k* × *m* − 0.25 = 70 × 0.3 − 0.25 = 95.

Reference animal dose (mg/kg)/MRref = 14.28/24 = ∼0.595.

Target animal dose (mg/kg)/MRref × MRtarget = 0.595 × 95 = 56 mg/kg for rats.

Where: MR = metabolic rate; *k* = constant of the taxonomic group (placental mammals = 70);*m* = body mass.

*(2) Experimental Design*. Eight male Wistar-Kyoto rats and forty male SHRs were randomly divided into six experimental groups (*n* = 8), as follows: (1) negative control (NC; SHR treated with vehicle (filtered water); 0.1 mL/100 g *plus* nicotine 1.8 mg/kg); (2) EESM 14 (SHR treated with EESM 14 mg/kg *plus* nicotine 1.8 mg/kg); (3) EESM 28 (SHR treated with EESM 28 mg/kg *plus* nicotine 1.8 mg/kg); (4) EESM 56 (SHR treated with EESM 56 mg/kg *plus* nicotine 1.8 mg/kg); (5) HCTZ (positive control; SHR treated with hydrochlorothiazide 25 mg/kg *plus* nicotine 1.8 mg/kg); and (6) naïve (WK rats treated only with the vehicle 0.1 mL/100 g). All treatments were per oral route, once a day, for 28 days. Nicotine administration followed the protocols developed by Bui et al. [13].

*(3) Renal Function*. On days 1 and 28, all rats were treated orally with NaCl 0.9% (5 mL/100 g) to impose hydration uniformity. Then, the animals were placed individually in metabolic cages. Urine samples were collected over 24 hours, and their volumes were registered. Urine pH and density were determined by a digital pH meter (Q400MT; Quimis Instruments, Brazil) and handheld refractometer (NO107; Nova Instruments, Brazil), respectively. Urinary sodium, potassium, chloride, urea, and creatinine levels were quantified using an automated biochemical analyzer (Cobas Integra 400 plus, Roche).

*(4) Electrocardiography (ECG)*. On the twenty-ninth day, all animals were subjected to ECG. Initially, the rats were anesthetized (ketamine 100 mg/kg plus xylazine 20 mg/kg; intramuscularly), and electrodes were positioned on two forelimbs and hindlimbs. After 5 minutes, the segments (ms; PR, QRS, QT, and QTc) and wave amplitudes (mV; *P*, *Q*, *R*, and *S*) were recorded. The results were recorded using a 12-lead ECG recorder (WinCardio, Micromed, Brasília, Brazil).

*(5) Blood Pressure (BP) and Heart Rate (HR)*. After the ECG recording, the animals received 30 IU heparin by subcutaneous route. Then, the left carotid artery was then isolated, cannulated, and connected to a pressure transducer attached to a PowerLab® recording system (Chart, v 7.1; ADI Instruments, Castle Hill, Australia). The HR, systolic (SBP) blood pressure, diastolic blood pressure (DBP), and mean arterial pressure (MAP) were registered for 20 min.

*(6) Biochemical Analysis*. After BP and HR recording, the arterial blood samples were collected and centrifuged (1000 g for 10 min) to obtain serum. The potassium, sodium, creatinine, and urea levels were measured by an automated analyzer (Roche® Cobas Integra 400 plus). Adrenaline, aldosterone, and nitrotyrosine (NT) concentrations were determined by enzyme-linked immunosorbent assay (ELISA; BD Biosciences, CA, USA). Malondialdehyde (MDA) levels were determined by the thiobarbituric acid reactive substances assay (Cayman Chemical, Ann Arbor, Michigan, USA). Plasma nitrite concentrations were measured according to Schmidt et al. [14]. The serum angiotensin-converting enzyme (ACE) activity was determined by indirect fluorimetry [15].

*(7) Peripheral Arterial Reactivity*. The mesenteric vascular beds (MVBs) were removed and prepared according to McGregor's [16] methods. The preparations were attached to a perfusion system which was constantly perfused with PSS (composition in mM: NaCl 119; KCl 4.7; CaCl_2_ 2.4; MgSO_4_ 1.2; NaHCO_3_ 25.0; KH_2_PO_4_ 1.2; dextrose 11.1; and EDTA 0.03) and gassed with 95% O_2_/5% CO_2_ at 37°C. After a stabilization period of 30 minutes, tissue integrity was analyzed through the response of a bolus injection of KCl (120 mmol). Afterwards, different dosages of phenylephrine (Phe; 1, 2, 10, and 30 nmol) and endothelin-1 (ET-1; 1, 2, 10, and 30 nmol) were administered in MVBs. The organs underwent another 30 minutes for stabilization while continuously infused with PSS plus 3 *µ*M Phe. After the contractile process was stabilized, the vascular reactivity of acetylcholine (ACh; 10, 30, 100, and 300 pmol) and sodium nitroprusside (SNP; 10, 30, 100, and 300 pmol) was evaluated. The changes in perfusion pressure (PP; mm Hg) were detected by a pressure transducer connected to a PowerLab® recording system and its application software (Chart, v 7.1; ADI Instruments, Castle Hill, Australia). At the end of the experiments, all animals were euthanized by isoflurane overdose.

*(8) Myocardial Function Indices*. After collecting mesenteric vascular beds (MVBs), the hearts were quickly removed and cooled in 0.9% NaCl solution. The hearts were mounted on a Langendorff apparatus and perfused (at a pressure of 70 mm·Hg) with Krebs and Ringer's bicarbonate solution (mmol/L: NaCl, 120; HNaCO_3_, 25; KCl, 4.8; MgSO_4_, 1.33; KH_2_PO_4_, 1.2; CaCl_2_, 1.6; Na_2_ EDTA, 0.02; and glucose, 10). The perfusate was carbonated with 95% O_2_ and 5% CO_2_ (pH 7.4; 37°C). Then, the atrium was removed, and a latex balloon was inserted into the left ventricle. The balloon volume was adjusted to a left ventricular end-diastolic pressure of 10 mm Hg. The following parameters were registered by the PowerLab® recording system (Chart, v 7.1; ADI Instruments, Castle Hill, Australia): left ventricular developed pressure (LVDP; peak systolic pressure minus end-diastolic pressure), peak rate of contraction (+d*p/*d*t*_max_), peak rate of relaxation (−d*p/*d*t*_min_), and the rate pressure product (RPP = LVDP × HR).

*(9) Relative Organ Weight, Histopathology, and Morphometry*. At the end of the experiments, the kidneys, liver, and heart were removed and cleaned, and the relative weight was determined (RW% = absolute organ weight × 100/body weight). Then, heart fragments were fixed in 10% buffered formalin, cleaved, dehydrated with increasing ethanol concentrations, diaphonized in xylol, and embedded in paraffin. Sections were then cut at a thickness of 4 *μ*m and stained with hematoxylin and eosin (HE). The heart area and the right (RV) and left (LV) ventricles and interventricular septum (IS) thickness were measured. All images were obtained and evaluated using Motic Images Plus 2.0 software.

#### 2.3.4. Investigation of the Role of the Endothelin and Renin-Angiotensin System on the Cardioprotective Effects of *S. microglossa* Extract

Different groups of hypertensive rats (SHRs 28 days nicotine-treated; *n* = 8) were initially anesthetized and prepared for direct blood pressure measurement, as described above. After cannulation and MAP stabilization, the effects of angiotensin I (Ang I; 10 pmol/kg, i.v.), angiotensin II (Ang II; 10 pmol/kg, i.v.), and ET-1 (10 pmol/kg, i.v.) on the arterial pressure were recorded. After returning blood pressure levels to baseline, a bolus dose of EESM (56 mg/kg) was administered intraduodenally. After 30 minutes, a new dose of angiotensin I, angiotensin II, and ET-1 was administered. SBP, DBP, and MAP values were registered for 20 min.

### 2.4. Statistical Analysis

The results are shown as mean ± standard error of the mean (SEM) of 8 rats or preparations per group. Statistical analyses were performed by one-way analysis of variance (ANOVA) followed by Tukey's test. A *p* value less than 0.05 was considered statistically significant.

## 3. Results

### 3.1. Acute Toxicity

No deaths were detected during the 14-day study, allowing to suggest an LD50 as greater than 2,000 mg/kg. We identified no clinical or behavioral signs indicating EESM toxicity, including body weight and food consumption. Furthermore, no significant histopathological changes were observed (data not shown).

### 3.2. Effects on Kidney Function

The values that show urinary parameters of all experimental groups on the first and twenty-eighth day of treatment are shown in Tables 1 and 2. On the first day of treatment, all 6-month-old nicotine-treated hypertensive rats showed a significant reduction in urinary volume and renal excretion of sodium and creatinine. On the other hand, after 28 days of treatment, all animals treated with the highest dose of EESM (56 mg/kg) or with HCTZ reversed this change, showing values similar to naïve animals.

### 3.3. Effects on Cardiac Electrical Activity

The effects of oral treatment with EESM and HCTZ on electrocardiographic parameters are shown in Table 3. A significant increase in the QT and QTc segments and the *R* wave amplitude was observed in the NC, EESM (14 and 28 mg/kg), and HCTZ groups. On the other hand, the animals in the EESM 56 mg/kg group did not show any significant electrocardiographic alterations, with values similar to those found in the naïve rats.

### 3.4. Effects on Blood Pressure

The effects of oral treatment with EESM and HCTZ on blood pressure values are shown in Table 4. Rats in the NC group showed a significant increase in SBP, DBP, MAP, and HR compared to naïve animals. Treatment with EESM at its highest dose (56 mm/kg) or with HCTZ prevented this change, maintaining blood pressure levels similar to those in the naïve rats. Similarly, treatment with EESM 56 mg/kg or HCTZ also significantly reduced the HR of nicotine-treated hypertensive rats.

### 3.5. Effects on Biochemical Parameters

The effects of oral treatment with EESM and HCTZ on biochemical parameters are shown in Table 5. Serum creatinine levels were significantly increased in animals from the NC and EESM 14 and 28 mg/kg groups. Treatment with HCTZ and EESM at its highest dose (56 mg/kg) reduced this parameter, maintaining creatinine values similar to those found for naïve animals. NT and MDA serum levels were significantly elevated, and nitrite levels were reduced in nicotine-treated hypertensive rats that received vehicle (NC), HCTZ, and EESM at 14 and 28 mg/kg. Treatment with EESM at a dose of 56 mg/kg maintained levels of these oxidative stress markers similar to those found in naïve rats. Serum adrenaline levels were significantly increased in nicotine-treated hypertensive rats compared to naïve animals. None of the treatments significantly reduced adrenaline serum levels in hypertensive animals. All other evaluated parameters were not altered in any of the experimental groups.

### 3.6. Effects on Mesenteric Arterial Reactivity

The effects of oral treatment with EESM and HCTZ on mesenteric arterial reactivity are shown in Table 6. Mesenteric vascular reactivity to ACh and Phe was significantly altered in nicotine-treated rats treated with vehicle alone (NC). On the other hand, treatment for 28 days with HCTZ or with EESM at its highest dose was able to reverse this change, maintaining arterial responsiveness similar to that observed in naïve animals. Vascular reactivity to the SNP was not altered in any experimental group.

### 3.7. Effects on Hemodynamics and Cardiac Structure

**Figure 1:**
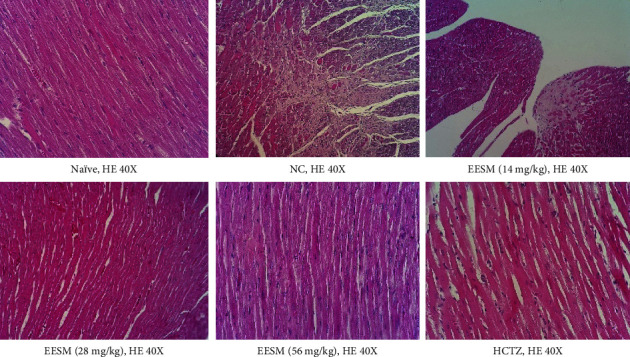
Representative images of the left ventricle of nicotine-treated hypertensive rats that received the EESM for 28 days. The black arrow represents an area of fibrous connective tissue deposition (scar). The black circle delimits an extensive area of fibrous tissue deposition associated with necrosis of cardiac fibers. EESM: hypertensive rats that received three different doses of the *Solidago microglossa* extract; HCTZ: hypertensive animals that received daily doses of hydrochlorothiazide (25 mg/kg); HE: hematoxylin and eosin stain; NC: hypertensive animals that received daily doses of the vehicle; Naïve: normotensive animals that received only the vehicle.

The effects of oral treatment with EESM and HCTZ on hemodynamics and cardiac structure are shown in Figure 1 and Table 7. In the left ventricle myocardium of the animals in the NC group, it was possible to visualize an extensive area of fibrous tissue deposition associated with discreet necrosis and mononuclear cell inflammatory infiltrate. Hypertensive animals treated with the lowest dose of EESM (14 mg/kg) also showed a discrete focal area of fibrosis in the subendocardial layer of the myocardium. All other experimental groups did not show significant histopathological alterations.

All hypertensive animals that were treated with the vehicle alone showed a significant reduction in ventricular function, including the LVDP (mm Hg), +dp/dt_max_ (mm·Hg/s), −dp/dt_min_ (mm·Hg/s), and RPP (mm·Hg-beats/min). In addition, a significant increase in the relative weight of the heart was evidenced, directly influenced by the thickening of the left ventricular wall. Only hypertensive animals treated with HCTZ or EESM at its highest dose (56 mg/kg) showed normalization of these alterations, showing values similar to those observed in naïve animals.

### 3.8. Effects on Endothelin and Renin-Angiotensin System

The effects of intraduodenal administration of EESM (25 mg/kg) on endothelin and renin-angiotensin system are presented in Table 8. Before intraduodenal administration of EESM, intravenous administration of Ang I, Ang II, and ET-1 significantly increased SBP, DBP, and MAP levels compared with baseline blood pressure values. Treatment with EESM prevented the increase in blood pressure levels caused by the administration of ET-1, while the effects of Ang I and II remained unaltered.

## 4. Discussion

Smoking is widespread in all societies in the world. Despite addiction, it is agreed that chronic smoking affects the function and structure of the cardiovascular system [17]. Among the components of tobacco, nicotine is the most significant contributor to the genesis and progression of CVDs, including vascular diseases and cardiomyopathies [18]. Nicotine causes endothelial dysfunction and stimulates the oxidative and inflammatory cascade that leads to the development of atherosclerosis and hypertension. Furthermore, it can aggravate preexisting hypertension and induce coronary spasms, precipitating a myocardial infarction. Moreover, nicotine increases free radical production, vascular wall adhesion, and plasma reduction in fibrinolytic activity [13]. One of the main mechanisms that involve these effects is related to the increase in the release of catecholamines in the sympathetic nerve endings and the channel ion modulations [19]. Thus, this work aimed to investigate the effects of a Brazilian pharmacopeial herbal preparation on SHRs treated with nicotine. Therefore, we aim to assess how much the EESM contributed to reducing the renal, cardiac, and vascular dysfunction that may be associated with nicotine-treated hypertensive rats.

Although the hemodynamic and morphological alterations that hypertension induces in rodents are already well known, we used two different experimental conditions. First, we used 6-month-old SHRs, stabilizing hypertension and leading to cardiac and vascular morphological changes [20]. In addition, we used nicotine to potentialize cardiovascular changes, hypothesizing that EESM could induce cardioprotective effects against these conditions. As expected, nicotine treatment in 6-month-old SHRs caused a significant reduction in renal function, with lower urinary volume and renal elimination of sodium and creatinine. In addition, serum markers of the redox state and blood pressure levels remained significantly elevated, contributing to changes in vascular reactivity and reduced ventricular function.

After 28 days of treatment, the highest dose of EESM could mitigate the renal and cardiovascular changes induced in the nicotine-treated hypertensive rats. Physiopathological alterations observed in the animals that received only the vehicle were completely reversed in the rats treated with the EESM, maintaining cardiovascular conditions similar to those found in the naïve animals. Although the results were interesting, the mechanisms by which the EESM produced these responses remained unclear. The EESM was standardized with 1% quercetin-3-rhamnoside, a glycosylated flavonoid. It is now known that glycosylated flavonoids have better solubility and oral absorption than isolated aglycone, e.g., quercetin [21]. Nevertheless, it is already widely known that quercetin has significant antioxidant, vasodilator, antihypertensive, and cardioprotective activity [22]. Data showed that quercetin inhibits vascular superoxide production induced by ET-1 and increases nitric oxide levels [23, 24]. Furthermore, quercetin induces oxidative and endoplasmic reticulum stress suppression via ET-1/MAPK signalling [25]. Although the information about aglycone is promising, its glycosylated form, i.e., quercetin-3-rhamnoside, needs investigation.

We hypothesize that the effects of quercetin-3-rhamnoside and, consequently, of the EESM could be similar to those described for quercetin. Thus, as the extract significantly affected lipid peroxidation and tyrosine nitration mediated by reactive nitrogen species, the NO bioavailability was increased, reverberating into a significant antioxidant effect. Together, this result directly contributed to a vasodilator response and blood pressure reduction, helping to maintain the integrity of the vascular endothelium [26, 27]. On the other hand, as vascular reactive oxygen species (ROS) production can be induced by ET-1 and Ang II [28, 29], we chose to investigate the role of the renin-angiotensin and endothelin system in the hypotensive response of the EESM because it is known that the direct inhibition of the action of ET-1 can prevent the effects of Ang II from stimulating cardiac hypertrophy [28]. The treatment with EESM (at its highest dose) prevented ET1-induced hypertension without affecting the renin-angiotensin system, suggesting the direct role of the endothelin signalling pathway.

Endothelins are vasoconstrictor peptides represented by ET-1, ET-2, and ET-3. Only ET-1 is produced by endothelial cells and is related to hemodynamic disorders and endothelial dysfunction. Stimuli such as ischemia, hypoxia, or shear stress induce the transcription of messenger RNA, with consequent synthesis and secretion of ET-1. On the other hand, ET-1 interacts with several hormones, vasoactive peptides, and growth factors, increasing vascular shear stress [30]. Smokers have increased ET-1 levels due to nicotine-stimulated production by endothelial cells [31]. Furthermore, it was described that the antagonism of endothelin ET(A) receptors attenuated the increase in blood pressure after nicotine injections, suggesting that ET-1 may have a critical role in the effects of nicotine [32]. Thus, a limitation of our study was not being able to relate the effects of EESM with the blockade of ETA receptors.

In summary, the data obtained in this study allow us to conjecture that the cardioprotective effects of EESM are likely due to antioxidant effects and ET-1 inhibitors. We cannot claim that the pharmacological effects presented here are only due to quercetin-3-rhamnoside. Still, we believe it is an important agent that acts synchronously with other secondary metabolites in the EESM.

## 5. Conclusion

This study presented EESM as a possible cardioprotective drug that prevents cardiovascular dysfunctions in nicotine-treated hypertensive rats. Our data suggest EESM as a potential adjuvant therapy when cardioprotective effects are required.

## Figures and Tables

**Table 1:** Effects of oral treatment with *Solidago microglossa* extract (EESM) and hydrochlorothiazide (HCTZ) on urinary parameters on the 1^st^ day of treatment.

Parameter	Naïve	NC	HCTZ	EESM (14 mg/kg)	EESM (28 mg/kg)	EESM (56 mg/kg)
Urinary volume (mL/100 g/24 h)	4.71 ± 0.42	2.32 ± 0.27^a^	2.00 ± 0.28^a^	2.59 ± 0.45^a^	2.40 ± 0.35^a^	2.35 ± 0.31^a^
pH	7.71 ± 0.26	7.73 ± 0.11	7.72 ± 0.25	7.73 ± 0.35	7.72 ± 0.20	7.74 ± 0.20
Density	1037 ± 5.53	1038 ± 2.09	1039 ± 5.54	1037 ± 3.87	1036 ± 5.32	1037 ± 5.32
Chloride (*µ*mol/100 g/24 h)	1011.34 ± 213.73	1034.22 ± 193.21	1055.64 ± 199.66	1034.13 ± 155.89	1048.35 ± 169.03	1023.22 ± 134.12
Potassium (*µ*mol/100 g/24 h)	875.11 ± 112.21	765.17 ± 102.25	815.21 ± 99.77	746.55 ± 97.21	793.51 ± 101.77	781.11 ± 104.32
Sodium (*µ*mol/100 g/24 h)	1432.12 ± 224.55	933.44 ± 87.66^a^	875.26 ± 99.77^a^	921.15 ± 80.21^a^	885.25 ± 94.44^a^	875.77 ± 90.91^a^
Urea (mg/100 g/24 h)	174.30 ± 22.11	166.55 ± 21.77	154.66 ± 33.54	168.55 ± 31.47	184.52 ± 39.99	171.58 ± 28.24
Creatinine (mg/100 g/24 h)	2.17 ± 0.31	1.21 ± 0.29^a^	1.37 ± 0.27^a^	1.40 ± 0.21^a^	1.27 ± 0.25^a^	1.30 ± 0.24^a^

Statistical analyses were performed using one-way ANOVA followed by Tukey's test. Values are expressed as mean ± SEM (standard error of the mean) of 8 animals per group. ^a^*p* < 0.05 when compared to the naïve rats. EESM: hypertensive rats that received three different doses of the *Solidago microglossa* extract; HCTZ: hypertensive animals that received daily doses of hydrochlorothiazide (25 mg/kg); NC: hypertensive animals that received daily doses of the vehicle; Naïve: normotensive animals that received only the vehicle.

**Table 2:** Effects of oral treatment with *Solidago microglossa* extract (EESM) and hydrochlorothiazide (HCTZ) on urinary parameters on the 28^th^ day of treatment.

Parameter	Naïve	NC	HCTZ	EESM (14 mg/kg)	EESM (28 mg/kg)	EESM (56 mg/kg)
Urinary volume (mL/100 g/24 h)	4.46 ± 0.56	2.06 ± 0.46^a^	4.01 ± 0.50^bcd^	2.33 ± 0.31^a^	2.81 ± 0.32^a^	4.77 ± 0.66^bcd^
pH	7.25 ± 0.19	7.32 ± 0.12	7.24 ± 0.17	7.31 ± 0.18	7.20 ± 0.13	7.21 ± 0.13
Density	1022 ± 2.90	1024 ± 2.34	1023 ± 2.39	1024 ± 2.50	1023 ± 2.45	1022 ± 2.45
Chloride (*µ*mol/100 g/24 h)	938.34 ± 77.01	825.55 ± 54.33	899.67 ± 49.88	831.24 ± 77.62	903.22 ± 89.31	912.36 ± 74.34
Potassium (*µ*mol/100 g/24 h)	645.33 ± 67.33	621.44 ± 71.29	604.33 ± 71.12	633.12 ± 69.38	618.21 ± 69.21	633.41 ± 70.11
Sodium (*µ*mol/100 g/24 h)	1524.44 ± 198.33	912.33 ± 74.33^a^	1324.33 ± 87.21^bcd^	833.43 ± 79.54^a^	899.44 ± 79.21^a^	1477.61 ± 121.22^bcd^
Urea (mg/100 g/24 h)	166.21 ± 19.33	152.77 ± 22.69	149.12 ± 33.21	159.12 ± 30.12	162.66 ± 29.45	148.44 ± 31.22
Creatinine (mg/100 g/24 h)	1.96 ± 0.32	1.16 ± 0.18^a^	1.88 ± 0.31^bcd^	1.04 ± 0.23^a^	1.10 ± 0.29^a^	2.05 ± 0.19^bcd^

Statistical analyses were performed using one-way ANOVA followed by Tukey's test. Values are expressed as mean ± SEM (standard error of the mean) of 8 animals per group. ^a^*p* < 0.05 when compared to the naïve rats. ^b^*p* < 0.05 when compared to the NC group. ^c^*p* < 0.05 when compared to the EESM 14 mg/kg. ^d^*p* < 0.05 when compared to the EESM 28 mg/kg. EESM: hypertensive rats that received three different doses of the *Solidago microglossa* extract; HCTZ: hypertensive animals that received daily doses of hydrochlorothiazide (25 mg/kg); NC: hypertensive animals that received daily doses of the vehicle; Naïve: normotensive animals that received only the vehicle.

**Table 3:** Effects of oral treatment with *Solidago microglossa* extract (EESM) and hydrochlorothiazide (HCTZ) on electrocardiographic parameters.

Parameter	Naïve	NC	HCTZ	EESM (14 mg/kg)	EESM (28 mg/kg)	EESM (56 mg/kg)
*Segments (ms)*						
PR	79.80 ± 7.06	79.50 ± 2.44	80.25 ± 2.77	82.29 ± 5.44	81.75 ± 6.40	80.66 ± 4.12
QRS	69.40 ± 2.87	70.13 ± 1.80	71.88 ± 1.80	74.86 ± 8.18	72.25 ± 3.94	71.34 ± 3.01
QT	140.6 ± 10.76	184.6 ± 5.59^a^	170.1 ± 6.04^a^	179.8 ± 10.66^a^	177.8 ± 10.66^a^	142.7 ± 7.46^bcde^
QTc	271.2 ± 21.53	367.9 ± 14.16^a^	329.4 ± 14.15^a^	343.6 ± 29.56^a^	340.3 ± 27.35^a^	290.7 ± 20.77^bcde^

*Waves (mV)*						
*P*	0.056 ± 0.009	0.059 ± 0.012	0.055 ± 0.007	0.054 ± 0.007	0.058 ± 0.008	0.056 ± 0.009
*Q*	−0.010 ± 0.006	−0.014 ± 0.005	−0.015 ± 0.003	−0.011 ± 0.005	−0.010 ± 0.006	−0.012 ± 0.007
*R*	0.30 ± 0.02	0.39 ± 0.02^a^	0.37 ± 0.02^a^	0.40 ± 0.02^a^	0.41 ± 0.03^a^	0.31 ± 0.02^bcde^
*S*	−0.076 ± 0.02	−0.074 ± 0.02	−0.076 ± 0.02	−0.077 ± 0.02	−0.075 ± 0.02	−0.074 ± 0.02

Statistical analyses were performed using one-way ANOVA followed by Tukey's test. Values are expressed as mean ± SEM (standard error of the mean) of 8 animals per group. ^a^*p* < 0.05 when compared to the naïve rats. ^b^*p* < 0.05 when compared to the NC group. ^c^*p* < 0.05 when compared to the EESM 14 mg/kg. ^d^*p* < 0.05 when compared to the EESM 28 mg/kg. ^e^*p* < 0.05 when compared to the HCTZ. EESM: hypertensive rats that received three different doses of the *Solidago microglossa* extract; HCTZ: hypertensive animals that received daily doses of hydrochlorothiazide (25 mg/kg); NC: hypertensive animals that received daily doses of the vehicle; Naïve: normotensive animals that received only the vehicle.

**Table 4:** Effects of oral treatment with *Solidago microglossa* extract (EESM) and hydrochlorothiazide (HCTZ) on blood pressure values.

Parameter	Naïve	NC	HCTZ	EESM (14 mg/kg)	EESM (28 mg/kg)	EESM (56 mg/kg)
SBP (mm·Hg)	114 ± 7.33	154 ± 5.44^a^	111 ± 2.89^bcd^	155 ± 3.95^a^	147 ± 3.45^a^	117 ± 7.11^bcd^
DBP (mm·Hg)	76.4 ± 4.04	103.3 ± 4.97^a^	80.3 ± 3.28^bcd^	105.1 ± 3.65^a^	99.8 ± 3.43^a^	78.4 ± 3.12^bcd^
MAP (mm·Hg)	92.2 ± 3.69	122.2 ± 4.01^a^	96.5 ± 3.97^bcd^	118.1 ± 3.30^a^	117.0 ± 4.01^a^	90.8 ± 3.55^bcd^
HR (bpm)	237 ± 15.3	394 ± 20.72^a^	302 ± 17.53^abcd^	378 ± 21.66^a^	376 ± 23.30^a^	297 ± 20.3^abcd^

Statistical analyses were performed using one-way ANOVA followed by Tukey's test. Values are expressed as mean ± SEM (standard error of the mean) of 8 animals per group. ^a^*p* < 0.05 when compared to the naïve rats. ^b^*p* < 0.05 when compared to the NC group. ^c^*p* < 0.05 when compared to the EESM 14 mg/kg. ^d^*p* < 0.05 when compared to the EESM 28 mg/kg. EESM: hypertensive rats that received three different doses of the *Solidago microglossa* extract; HCTZ: hypertensive animals that received daily doses of hydrochlorothiazide (25 mg/kg); NC: hypertensive animals that received daily doses of the vehicle; Naïve: normotensive animals that received only the vehicle. DBP: diastolic blood pressure; HR: heart rate; MAP: mean arterial pressure; SBP: systolic blood pressure.

**Table 5:** Effects of oral treatment with *Solidago microglossa* extract (EESM) and hydrochlorothiazide (HCTZ) on biochemical parameters.

Parameter	Naïve	NC	HCTZ	EESM (14 mg/kg)	EESM (28 mg/kg)	EESM (56 mg/kg)
Sodium (mmol/L)	115.1 ± 10.3	113.4 ± 12.2	110.7 ± 13.4	108.8 ± 11.9	112.8 ± 15.5	114.5 ± 14.4
Potassium (mmol/L)	5.23 ± 0.22	5.36 ± 0.30	5.33 ± 0.26	5.40 ± 0.20	5.31 ± 0.21	5.22 ± 0.24
Creatinine (mg/dL)	0.87 ± 0.22	1.91 ± 0.25^a^	1.11 ± 0.18^bcd^	1.88 ± 0.17^a^	1.71 ± 0.13^a^	0.97 ± 0.18^bcd^
Nitrite (*μ*M)	100.5 ± 10.77	62.4 ± 8.31^a^	77.5 ± 6.22^a^	80.3 ± 10.21	88.2 ± 7.31	110.1 ± 8.14^bcde^
NT (*μ*mol/L)	0.011 ± 0.006	0.051 ± 0.009^a^	0.044 ± 0.008^a^	0.035 ± 0.008	0.030 ± 0.009	0.010 ± 0.005^bcde^
MDA (mmol/L)	7.2 ± 2.3	16.6 ± 2.33^a^	15.6 ± 2.33^a^	13.7 ± 1.6^a^	12.7 ± 3.5^a^	6.2 ± 2.25^bcde^
Adrenaline (pg/mL)	0.72 ± 0.09	1.40 ± 0.11^a^	1.41 ± 0.19^a^	1.38 ± 0.17^a^	1.41 ± 0.15^a^	1.39 ± 0.16^a^
Aldosterone (pg/mL)	120.4 ± 12.21	122.5 ± 10.88	125.5 ± 13.12	131.6 ± 11.43	130 ± 12.44	129 ± 10.31
ACE activity (nmol/min/ml)	80.1 ± 6.33	87.5 ± 7.21	90 ± 8.33	85.55 ± 8.21	83.37 ± 7.34	91.22 ± 12.77

Statistical analyses were performed using one-way ANOVA followed by Tukey's test. Values are expressed as mean ± SEM (standard error of the mean) of 8 animals per group. ^a^*p* < 0.05 when compared to the naïve rats. ^b^*p* < 0.05 when compared to the NC group. ^c^*p* < 0.05 when compared to the EESM 14 mg/kg. ^d^*p* < 0.05 when compared to the EESM 28 mg/kg. ^e^*p* < 0.05 when compared to the HCTZ. EESM: hypertensive rats that received three different doses of the *Solidago microglossa* extract; HCTZ: hypertensive animals that received daily doses of hydrochlorothiazide (25 mg/kg); NC: hypertensive animals that received daily doses of the vehicle; Naïve: normotensive animals that received only the vehicle. ACE: angiotensin-converting enzyme; MDA: malondialdehyde; NT: nitrotyrosine.

**Table 6:** Effects of oral treatment with *Solidago microglossa* extract (EESM) and hydrochlorothiazide (HCTZ) on mesenteric arterial reactivity.

Parameter	Naïve	NC	HCTZ	EESM (14 mg/kg)	EESM (28 mg/kg)	EESM (56 mg/kg)
*Phe (nmol)*						
1	2.69 ± 0.55	5.01 ± 1.34^a^	3.62 ± 1.02^bcd^	8.96 ± 3.12^a^	8.91 ± 2.76^a^	3.41 ± 0.76^bcd^
3	3.41 ± 0.68	6.43 ± 1.49^a^	4.87 ± 1.13^bcd^	13.24 ± 3.28^a^	34.52 ± 7.61^a^	4.56 ± 0.88^bcd^
10	17.36 ± 8.12	65.24 ± 19.10^a^	29.04 ± 12.11^bcd^	59.65 ± 16.96^a^	54.84 ± 50.31^a^	22.04 ± 7.11^bcd^
30	73.62 ± 9.98	156.61 ± 23.76^a^	87.64 ± 9.63^bcd^	184.30 ± 20.20^a^	146.50 ± 29.38^a^	82.33 ± 8.51^bcd^

*ACh (pmol)*						
10	−10.81 ± 3.39	−5.84 ± 2.93^a^	−13.56 ± 3.64^bcd^	−7.73 ± 2.57^a^	6.67 ± 1.67^a^	−14.21 ± 3.01^bcd^
30	−19.78 ± 3.09	−9.03 ± 2.67^a^	−17.15 ± 2.98^bcd^	−9.00 ± 3.84^a^	−10.38 ± 2.01^a^	−18.11 ± 2.44^bcd^
100	−23.52 ± 4.76	−12.89 ± 3.73^a^	−20.78 ± 3.95^bcd^	−12.54 ± 3.67^a^	−13.51 ± 3.25^a^	−23.43 ± 3.74^bcd^
300	−35.65 ± 5.46	−12.74 ± 3.76^a^	−30.83 ± 6.89^bcd^	−10.39 ± 3.82^a^	−13.31 ± 4.33^a^	−37.77 ± 7.21^bcd^

*SNP (pmol)*						
10	−4.11 ± 1.40	−5.25 ± 2.89	−5.53 ± 2.95	−6.20 ± 2.52	−5.57 ± 1.62	−5.11 ± 1.56
30	−6.57 ± 6.73	−6.43 ± 1.62	−7.89 ± 3.62	−7.87 ± 3.32	−8.97 ± 2.79	−6.88 ± 2.51
100	−8.01 ± 2.14	−7.00 ± 2.10	−9.53 ± 3.91	−8.75 ± 3.31	−10.13 ± 2.51	−9.99 ± 2.76
300	−17.64 ± 2.48	−15.99 ± 2.59	−16.34 ± 3.01	−17.56 ± 3.43	−18.12 ± 3.58	−16.34 ± 3.43

Statistical analyses were performed using one-way ANOVA followed by Tukey's test. Values are expressed as mean ± SEM (standard error of the mean) of 8 animals per group. ^a^*p* < 0.05 when compared to the naïve rats. ^b^*p* < 0.05 when compared to the NC group. ^c^*p* < 0.05 when compared to the EESM 14 mg/kg. ^d^*p* < 0.05 when compared to the EESM 28 mg/kg. EESM: hypertensive rats that received three different doses of the *Solidago microglossa* extract; HCTZ: hypertensive animals that received daily doses of hydrochlorothiazide (25 mg/kg); NC: hypertensive animals that received daily doses of the vehicle; Naïve: normotensive animals that received only the vehicle. ACh: acetylcholine; Phe: phenylephrine; SNP: sodium nitroprusside.

**Table 7:** Effects of oral treatment with *Solidago microglossa* extract (EESM) and hydrochlorothiazide (HCTZ) on hemodynamics and cardiac structure.

Parameter	Naïve	NC	HCTZ	EESM (14 mg/kg)	EESM (28 mg/kg)	EESM (56 mg/kg)
*Myocardial function indices*						
LVDP (mm Hg)	122.5 ± 12.11	79.3 ± 9.33^a^	131.4 ± 14.44^bcd^	83.4 ± 8.77^a^	87.8 ± 10.21^a^	128.5 ± 15.21^bcd^
+d*p*/d*t*_max_ (mm Hg/s)	2811 ± 255	1975 ± 195^a^	2644 ± 311^bcd^	1766 ± 203^a^	1866 ± 205^a^	2733 ± 344^bcd^
−d*p*/d*t*_min_ (mm Hg/s)	−1776 ± 221	−1033 ± 121^a^	−1521 ± 276^bcd^	−1125 ± 155^a^	−1144 ± 177^a^	−1677 ± 299^bcd^
RPP (mm Hg-beats/min)	35222 ± 2343	20673 ± 3295^a^	33642 ± 2454^bcd^	18576 ± 3356^a^	23777 ± 3376^a^	34654 ± 3123^bcd^

*Relative weight*						
Heart (%)	0.25 ± 0.04	0.48 ± 0.07^a^	0.27 ± 0.03^bcd^	0.51 ± 0.08^a^	0.40 ± 0.07^a^	0.30 ± 0.04^bcd^

*Cardiac morphometry*						
Heart area (mm)	71.82 ± 4.20	86.86 ± 3.52^a^	78.17 ± 4.44^bcd^	86.35 ± 3.82^a^	90.38 ± 6.02^a^	77.38 ± 6.02^bcd^
LV lumen area (mm)	8.31 ± 0.47	6.91 ± 0.59^a^	8.34 ± 0.22^bcd^	6.93 ± 0.37^a^	5.99 ± 0.55^a^	8.72 ± 0.25^bcd^
RV lumen area (mm)	1.94 ± 0.18	1.99 ± 0.15	1.96 ± 0.13	1.97 ± 0.11	1.91 ± 0.12	1.93 ± 0.12
LV wall thickness (mm)	3.93 ± 0.31	5.83 ± 0.34^a^	3.77 ± 0.37^bcd^	5.79 ± 0.36^a^	5.78 ± 0.23^a^	3.89 ± 0.29^bcd^
RV wall thickness (mm)	1.84 ± 0.11	1.94 ± 0.17	1.87 ± 0.16	1.79 ± 0.11	1.74 ± 0.12	1.74 ± 0.12
IS thickness (mm)	5.13 ± 0.45	5.22 ± 0.49	4.93 ± 0.46	4.77 ± 0.52	5.22 ± 0.61	5.22 ± 0.61

Statistical analyses were performed using one-way ANOVA followed by Tukey's test. Values are expressed as mean ± SEM (standard error of the mean) of 8 animals per group. ^a^*p* < 0.05when compared to the naïve rats. ^b^*p* < 0.05 when compared to the NC group. ^c^*p* < 0.05 when compared to the EESM 14 mg/kg. ^d^*p* < 0.05 when compared to the EESM 28 mg/kg. EESM: hypertensive rats that received three different doses of the *Solidago microglossa* extract; HCTZ: hypertensive animals that received daily doses of hydrochlorothiazide (25 mg/kg); NC: hypertensive animals that received daily doses of the vehicle; Naïve: normotensive animals that received only the vehicle. IS: interventricular septum; LV: left ventricle; LVDP: left ventricular developed pressure; RPP: rate pressure product; RV: right ventricle; +d*p*/d*t*_max_: peak contraction rate; −d*p*/d*t*_min_: peak relaxation rate.

**Table 8:** Effects of intraduodenal administration of *Solidago microglossa* extract (EESM 25 mg/kg) on endothelin and renin-angiotensin system.

Parameter	Baseline blood pressure	Before EESM administration	After EESM administration
*Angiotensin I*			
10 pmoL/kg i.v.			
SBP (mm·Hg)	150 0.6 ± 3.22	190.7 ± 10.52^a^	197.5 ± 10.21^a^
DBP (mm·Hg)	95.2 ± 4.01	129.7 ± 9.24^a^	125.1 ± 11.44^a^
MAP (mm·Hg)	114.5 ± 4.33	150.7 ± 10.28^a^	156.8 ± 9.33^a^

*Angiotensin II*			
10 pmoL/kg i.v.			
SBP (mm·Hg)	155.9 ± 5.21	220.8 ± 15.32^a^	221.7 ± 16.77^a^
DBP (mm·Hg)	96.5 ± 4.88	133.5 ± 14.77^a^	133.4 ± 15.44^a^
MAP (mm·Hg)	118.6 ± 5.24	155.5 ± 13.21^a^	157.8 ± 12.77^a^

*Endothelin*			
10 pmoL/kg i.v.			
SBP (mm·Hg)	149.6 ± 5.11	194.2 ± 8.33^a^	142.9 ± 4.21^b^
DBP (mm·Hg)	97.8 ± 5.24	120.3 ± 7.21^a^	95.1 ± 3.77^b^
MAP (mm·Hg)	119.8 ± 4.99	147.5 ± 7.77^a^	147.7 ± 9.21^b^

Statistical analyses were performed using one-way ANOVA followed by Tukey's test. Values are expressed as mean ± SEM (standard error of the mean) of 8 animals per group. ^a^*p* < 0.05 when compared to baseline. ^b^*p* < 0.05 when compared to before EESM administration. DBP: diastolic blood pressure; HR: heart rate; MAP: mean arterial pressure; SBP: systolic blood pressure.

## Data Availability

The quantitative data used to support the findings of this study are available from the corresponding author upon request.

## Abbreviations

ACE: Angiotensin-converting enzyme
ACh: Acetylcholine
Ang-I: Angiotensin I
Ang-II: Angiotensin II
ANOVA: Analysis of variance
BP: Blood pressure
CaCl_2_: Calcium chloride
CVDs: Cardiovascular diseases
DBP: Diastolic blood pressure
ECG: Electrocardiography
EDTA: Ethylenediaminetetraacetic acid
EESM: Ethanol extract of *Solidago microglossa* leaves
ETA: Endothelin receptor
ET-1: Endothelin 1
ET-2: Endothelin 2
ET-3: Endothelin 3
HCTZ: Hydrochlorothiazide
HE: Hematoxylin and eosin
HR: Heart rate
IS: Interventricular septum
KCl: Potassium chloride
KH_2_PO_4_: Potassium dihydrogen phosphate
LV: Left ventricle
LVDP: Left ventricular developed pressure
MAP: Main arterial pressure
MAPK: Mitogen-activated protein kinase
MDA: Malondialdehyde
MgSO_4_: Magnesium sulfate
MVB: Mesenteric vascular bed
NaCl: Sodium chloride
NaHCO_3_: Sodium bicarbonate
NC: Negative control
NO: Nitric oxide
NT: Nitrotyrosine
Phe: Phenylephrine
PP: Perfusion pressure
PSS: Physiologic saline solution
RNA: Ribonucleic acid
ROS: Reactive oxygen species
RPP: Rate pressure product
RV: Right ventricle
SEM: Standard error of the mean
SHRs: Spontaneously hypertensive rats
SNP: Sodium nitroprusside
UFGD: Federal University of Grande Dourados
WK: Wistar-Kyoto.
